# The diagnostic value of the combination of patient characteristics, history, and clinical shoulder tests for the diagnosis of rotator cuff tear

**DOI:** 10.1186/s13018-014-0070-y

**Published:** 2014-08-07

**Authors:** Derk A van Kampen, Tobias van den Berg, Henk Jan van der Woude, Rene M Castelein, Vanessa AB Scholtes, Caroline B Terwee, W Jaap Willems

**Affiliations:** 1 Department of Orthopaedic Surgery and Traumatology, Waterland Hospital, Waterlandlaan 250, Purmerend 1441 RN, The Netherlands; 2 Department of Orthopaedic Surgery and Traumatology, OLVG Hospital, Amsterdam 1091 AC, The Netherlands; 3 Department of Orthopaedic Surgery and Traumatology, University Medical Center Utrecht, Utrecht 3584 CX, The Netherlands; 4 Department of Epidemiology and Biostatistics and the EMGO institute for Health and Care Research, VU University Medical Center, Amsterdam 1081 HZ, The Netherlands; 5 Shoulder Unit, Lairesse Kliniek, Valeriusplein, Amsterdam 1075 BG, The Netherlands

**Keywords:** Rotator cuff tears, Diagnostic test, Prediction model, Physical examination, Sensitivity, Specificity, Shoulder, Diagnoses

## Abstract

**Background:**

It is unknown which combination of patient information and clinical tests might be optimal for the diagnosis of rotator cuff tears. This study aimed to determine the diagnostic value of nine individual clinical tests for evaluating rotator cuff tear and to develop a prediction model for diagnosing rotator cuff tear.

**Methods:**

This prospective cohort study included 169 patients with shoulder complaints. Patients who reported a previous shoulder dislocation were excluded from the analysis (*N* = 69). One experienced clinician conducted 25 clinical tests of which 9 are specifically designed to diagnose rotator cuff pathology (empty can, Neer, Hawkins-Kenney, drop arm, lift-off test, painful arc, external rotation lag sign, drop sign, infraspinatus muscle strength test). The final diagnosis, based on magnetic resonance arthrography (MRA), was determined by consensus between the clinician and a radiologist, who were blinded to patient information. A prediction model was developed by logistic regression analysis.

**Results and discussion:**

In this cohort, 38 patients were diagnosed with rotator cuff tears. The individual overall accuracy of the rotator cuff clinical tests was 61%–75%. After backward selection, the model determined that the most important predictors of rotator cuff tears were higher age and a positive Neer test. This internally validated prediction model had good discriminative ability (area under the receiver operating characteristic curve (AUC) = 0.73).

**Conclusion:**

Our results showed that individual clinical shoulder tests had moderate diagnostic value for diagnosing rotator cuff tear. Our prediction model showed improved diagnostic value. However, the prediction value is still relatively low, supporting a low threshold for additional diagnostic tests for the diagnosis of rotator cuff tears.

**Level of evidence:**

Study of diagnostic test: level I.

## Introduction

Shoulder disorders rank among the most prevalent musculoskeletal disorders. They can be caused by many different pathologies, each requiring their own specific surgical or non-surgical treatment. Diagnostically, the shoulder is one of the most complex joints, due to its multiple directional movements. Furthermore, direct observation of shoulder motion is obscured by the muscles [1]. Rotator cuff (RC)-related disorders are among the most important causes for visiting the orthopaedic outpatient clinic. Correct diagnosis is essential for selecting the appropriate treatment plan.

Our previous publication on the diagnostic value of history and clinical tests for traumatic anterior shoulder instability showed that with a good history and physical examination, we are very well capable to diagnose traumatic anterior shoulder instability without the use of additional imaging, like magnetic resonance arthrography (MRA) [2].

There are also several shoulder-specific clinical tests for diagnosing RC tears. However, a recent meta-analysis showed that data was lacking to support most clinical tests used for diagnosing RC tears; moreover, there is a need for high-quality studies to test the diagnostic performance of parameters from patient history and physical examinations [3]-[5]. It is unknown which combination of patient information and clinical tests might be optimal for the diagnosis of rotator cuff tears [5]. Therefore, it is difficult to diagnose RC tears based purely on patient history and physical examination, leading to increase use of other techniques for establishing the diagnosis, including magnetic resonance imaging (MRI), ultrasound scans, and diagnostic arthroscopy. However, these tests are time-consuming, expensive, and/or invasive; thus, they should be restricted as much as possible.

Murrell and Walton and Park et al. reported that the combination of individual clinical test results and patient age could lead to improved diagnostic value for diagnosing a RC tear [6],[7]. However, in those studies, the reference standard (arthroscopy) was not performed in all patients, which could lead to a verification bias [8]. Ideally, an arthroscopy could be performed in every new patient with a shoulder complaint in the outpatient clinic to determine the diagnosis. However, because it is invasive, this approach is not ethically justified. Therefore, in our view, the reference standard should be a MRA [9],[10].

It would be very useful to have a prediction model, which combined patient characteristics, history, and results from a few clinical tests, for predicting the probability of a RC tear in individual patients. For example, the Ottawa Ankle Rules comprise one of the most famous prediction models presently used in orthopaedic surgery [11].

The first objective of the present study was to estimate the diagnostic value of clinical tests for rotator cuff tears. The second objective was to develop a prediction model for predicting the diagnosis of a RC tear. We hypothesized that the combined use of patient characteristics, history, and clinical tests will improve the diagnostic value for RC tears.

## Material and methods

### Patients

This prospective cohort study included new patients with shoulder complaints, recruited consecutively between February 2009 and June 2012 at the orthopaedic outpatient clinic. Institutional approval was obtained by Institutional review board of the OLVG Hospital, and written, signed, informed consent was obtained from all participants. Exclusion criteria were previously diagnosed shoulder disorders, fractures, frozen shoulder, arthritis, and deficiencies in reading and understanding the Dutch language.

We also excluded patients with a history of shoulder instability. Based on our previous study, the history of a previous shoulder dislocation showed to be a very strong predictor for the diagnosis of traumatic anterior shoulder instability.

Furthermore, in clinical practice, a potential rotator cuff tear is expected in patients with general shoulder complaints, not in young patients who present with repetitive shoulder dislocations. Therefore, patients who reported a previous shoulder dislocation were excluded from the analyses in this study [2].

### Data collection

One experienced orthopaedic surgeon (WJW) performed 25 shoulder-specific clinical tests on all patients according to a standardised diagnostic protocol based on the original descriptions of the clinical tests. The clinical tests were performed in a fixed order in every patient.

Nine of these clinical tests are considered specific for the rotator cuff, and these were selected for evaluation in this study. The examiner was blinded to the imaging analyses.

In addition to performing the typical clinical patient history, all patients were asked to complete an online (web-based) questionnaire, which allowed a standardised evaluation of patient history. Therefore, our final cohort consisted of patients with general complaints of the shoulder and who, based on history information, never experienced a shoulder dislocation. In this group, the aim is to predict the chances of a rotator cuff tear. We used validated, patient-reported outcome measures (PROMs) to ensure that the questions were standardised. Two PROMS were administered: the Simple Shoulder Test and the Oxford Shoulder Score [12],[13]. Subsequently, all patients underwent MRA of the involved shoulder as a reference standard for the final diagnosis.

### Clinical examination tests for assessment of rotator cuff tears

The empty can test, also known as the Jobe test, was performed with the patient standing, the shoulder in 90° abduction in the scapular plane, and with full internal rotation [14]. The thumbs were pointing toward the floor. The patient maintained this position against downward resistance applied by the examiner. The test was considered positive when the patient demonstrated weakness or pain during the applied resistance. The empty can test was developed specifically for the evaluation of the supraspinatus tendon.

The Neer test was performed with the patient sitting or standing [15]. The ipsilateral scapula was stabilised with the examiner's hand, and the patient's arm was passively elevated forward. The test was considered positive when the patient experienced pain. In the original description, Neer advised giving an injection of lidocaine in the subacromial space to relieve pain. Due to time limitations in the orthopaedic outpatient setting, we decided not to give patients a lidocaine injection. This was comparable with common practice, and it was consistent with the study by Park et al. [7]. The Neer test was developed specifically for the evaluation of the supraspinatus tendon.

The Hawkins-Kennedy test was performed with the examiner facing the seated or standing patient [16]. The patient's arm was elevated forward at 90°, and the elbow was flexed at 90°. The test was considered positive when pain occurred with passive internal rotation. The Hawkins-Kennedy test was developed specifically for the evaluation of the supraspinatus tendon.

The drop arm test, also known as Codman's sign, was performed with the patient standing [17]. The patient was asked to abduct the arm fully and then to reverse the motion slowly, in the same arc. When the arm dropped suddenly, the test was considered positive. The drop arm test was developed specifically for the evaluation of the supraspinatus tendon.

The lift-off test, also known as the Gerber test, was performed with the patient standing [18]. The patient was asked to place his/her hand on his/her back for maximum internal rotation and then to lift their hand off their back. The test was considered positive when the patient was not able to perform this. The lift-off test was developed specifically for the evaluation of the subscapularis tendon.

The painful arc test was performed with the patient standing [19]. The patient was asked to elevate the arm actively in the scapular plane, until the arm was fully elevated, and then to let the arm down in the same arc. The test was considered positive when the patient demonstrated pain or reported a painful catching between 60° and 120° elevation. The painful arc test was developed specifically for the evaluation of the rotator cuff.

The external rotation lag sign (ERLS) was performed with the patient seated [20]. The elbow was passively flexed to 90°, and the examiner held the shoulder at 20° elevation (in the scapular plane), near maximal external rotation (i.e. maximum external rotation minus 5, to avoid elastic recoil in the shoulder). The patient was then asked to maintain the external rotation in elevation as the examiner released the wrist but maintained support of the limb at the elbow. The sign was considered positive when a lag or angular drop occurred. The ERLS was developed specifically for the evaluation of the supraspinatus and infraspinatus tendon.

The drop sign, also known as the infraspinatus drop sign, was similar to the ERLS, but the arm was held at 90° elevation (in the scapular plane) by the examiner instead of the 20° elevation [20]. The drop sign was developed specifically for the evaluation of the infraspinatus tendon.

The infraspinatus muscle strength test was performed with the patient standing or sitting [7]. The elbow was flexed at 90° and the arm adducted to the trunk in neutral rotation. The examiner applied an internal rotation force to the arm while the patient resisted. The test was considered positive when the patient demonstrated weakness compared to the other side. The infraspinatus muscle strength was developed specifically for the evaluation of the infraspinatus tendon.

### Imaging technique, MRA

As additional imaging for making the final diagnosis, MRI, MRA, and ultrasound (US) are options. All three perform well for full-thickness tears; however, for partial rotator cuff tears, MRA has the best sensitivity and specificity. This was shown in two recent meta-analyses [21],[22].

Each patient first received an intra-articular administration of 10 mL Omnipaque 300 (300 mg I/mL iohexol; GE Healthcare BV, Eindhoven, The Netherlands). A 10-mL mixture of 0.5 mL Omniscan (0.5 mmol/mL Gd-DTPA-BMA; GE Healthcare BV) was added to 100 mL of 0.9% saline. The patient then received 12 to 15 mL of this solution, delivered with an 18-gauge needle inserted into the glenohumeral joint under fluoroscopic guidance by either an anterior or a posterior approach. MRA images were acquired within 30 min after injection. Patients were instructed to immobilize the shoulder of interest after the injection and during MRA. Imaging was performed with either a 1.0 T unit (MR Systems NT Release 4.5; Philips Medical Systems, Best, The Netherlands) or a 1.5 T unit (MR Systems Intera, Release 9.0, Philips Medical Systems). The following sequences were performed: T1-weighted fast spin echo (FSE) with fat-selective presaturation in an axial plane, oblique coronal plane, and oblique sagittal plane; oblique coronal proton density; and T2-weighted FSE and T1-weighted FSE with fat-selective presaturation, with the shoulder in an abduction-external rotation (ABER) position.

### Reference standard

The diagnosis based on the MRA was defined as the reference standard. The MRA images were reviewed in random order, and the evaluators were blinded to the patient's personal details, clinical history, and symptoms. The final diagnosis was made in consensus by the orthopaedic surgeon (WJW) and a musculoskeletal radiologist (HJW). Both had more than 15 years experience in evaluating shoulder MRAs. In the case of no consensus, a second experienced musculoskeletal radiologist was available to make the final diagnosis. We chose a consensus diagnosis for the MRA because previous studies have shown inter-observer variability for detecting full thickness and partial tears in the RC [9],[10]. All potential diagnoses for shoulder complaints were made in accordance with standard radiologic criteria [23].

Specifically, for RC tears, we used the following criteria. A complete (full-thickness) tear, with or without retraction of tendon edges, was identified as a gap, with hyperintense fluid signal intensity equal to water on a T2 FSE, with or without fat suppression, that extended from the articular space to the subacromial space and/or a hyperintense signal intensity on T1-weighted, fat-suppressed, MRA images in the various planes. An incomplete, or partial, tear was identified as an incomplete tendon defect, either on the bursal side or the articular side, with a hyperintense fluid signal intensity that extended within, but did not traverse, the tendon. Both partial- and full-thickness tears were considered RC tears.

### PROMs

The following validated PROMs were used to standardise the history questions.

#### The simple shoulder test

The Simple Shoulder Test (SST) measures functional limitations in patients with shoulder complaints [12]. It consists of 12 questions with dichotomous response options. Scores were summarised to a total score, which ranged from 0 (worst) to 12 (excellent). The SST has been validated in Dutch patients with shoulder complaints [24]. Question 8 (weakness) was used as a potential predictor in our prediction model.

#### Oxford Shoulder Score

The Oxford Shoulder Score (OSS) measures functional limitations in patients with shoulder complaints [12]. It contains 12 items with 5 response options. Scores were summarised to a total score, which ranged from 12 (excellent) to 60 (worst). The OSS has been validated in Dutch patients with shoulder complaints [25]. Question 12 (night pain) was used as a potential predictor in our prediction model.

### Statistical analysis

All patients with any type of RC tear were allocated to the RC tear group. Patients that had other diagnoses in addition to the RC tear remained in the RC tear group. All patients without a RC tear, based on the MRA, were allocated to the no-RC tear group, independent of their final diagnosis. Sensitivity, specificity, positive predictive value (PPV), negative predictive value (NPV), positive likelihood ratio, negative likelihood ratio, and overall accuracy were calculated with a 2 × 2 table. We defined overall accuracy as the percentage of true results (both true positives and true negatives).

We used logistic regression to develop the prediction model. Based on clinical experience and a meta-analysis by Hegedus et al. [4], we chose seven candidate predictors prior to the data analysis. These included age, night pain (OSS question 12 was dichotomised to no night pain versus any night pain), weakness (SST question 8), and four clinical tests (empty can, Neer, ERLS, and the Hawkins-Kennedy test) [26]. Strong correlations between predictors were investigated to avoid multicollinearity.

A logistic regression model was built to select relevant predictors and to estimate the regression coefficients. This was performed with a backward selection strategy in the full, seven-predictor model. Predictors were deleted step by step from the model based on the highest *p* value, until a stopping rule was reached, based on Akaike's information criterion [26]. The final model consisted only of predictors with a *p* value below 0.157. When the selected predictor was treated as a continuous variable, like age, it was checked to determine whether there was a linear relation between the predictor and the outcome.

We assessed the diagnostic performance of our model by determining calibration and discrimination [27]. Calibration referred to the agreement between observed and predicted outcomes. The Hosmer and Lemeshow ‘goodness-of-fit’ test indicated whether the model was a good fit to the data. The discriminative ability of the prediction model was assessed by the area under the receiver operating characteristic curve (AUC) or the equivalent c (concordance) index [28]. The AUC has a value between 0.5 (no discriminative ability) and 1.0 (perfect discriminative ability). In general, a prediction model is considered good when the AUC is above 0.8 [29].

A prediction model was fitted to the dataset at hand, and therefore, it was prone to overoptimism in new patients. Overoptimism is particularly common in small datasets, where the number of (starting) predictors is large compared to the smallest outcome group. Internal validation can correct for some of this overfitting with the bootstrapping method [27],[30]. In this case, 500 bootstraps were performed and a shrinkage factor was calculated to penalise the regression coefficients. For guidance on this protocol, see Steyerberg et al. [31]. Analyses were performed with R version 2.14.2 (http://www.R-project.org).

## Results

### Patients

**Figure 1:**
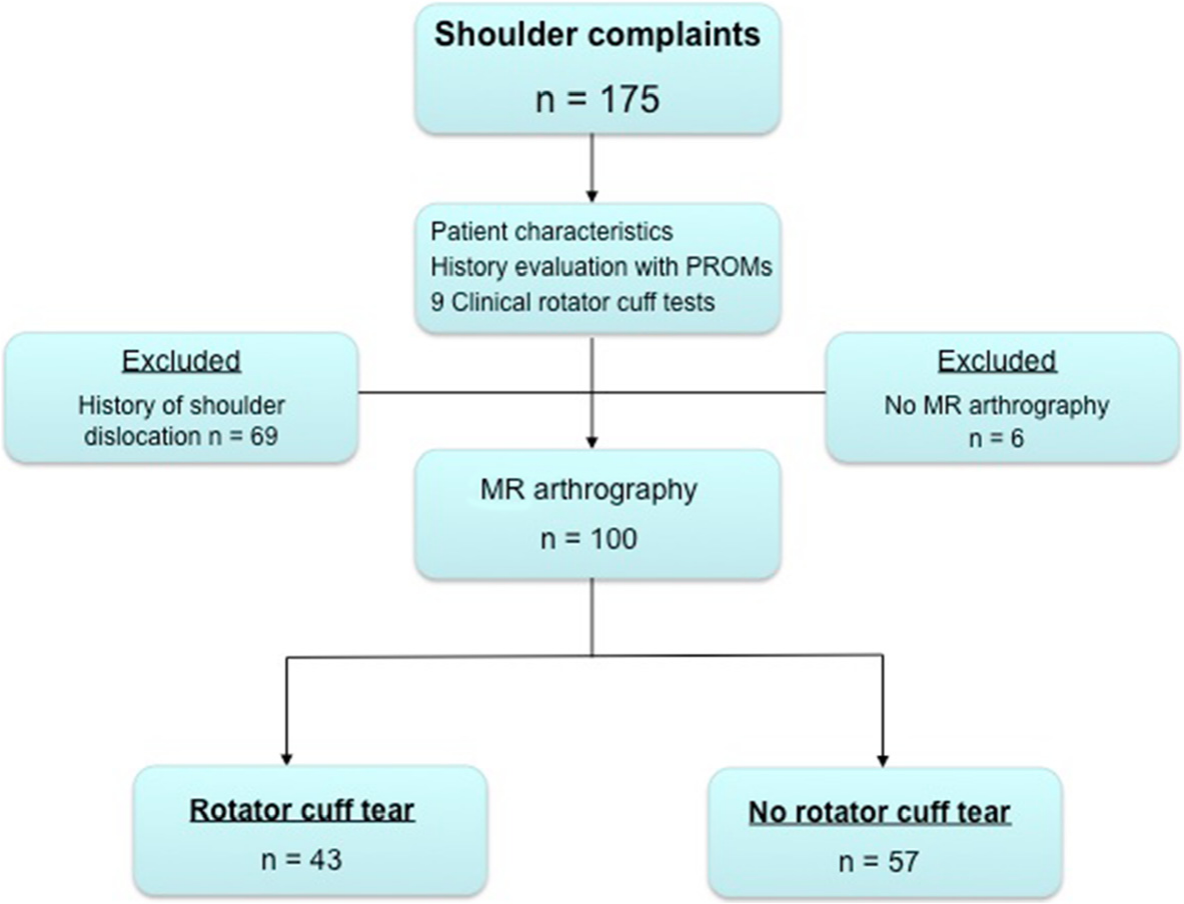
**The flow chart of the selection process.***n*, the number of shoulders evaluated.

The flow chart of the selection process for this study population is presented in Figure 1. The study included 174 new patients. One patient had complaints in both shoulders; thus, 175 shoulders were included. All patients completed the 25 clinical tests of which 9, specifically for the rotator cuff, were evaluated in this study. Six patients were lost to follow up: one patient went to another hospital, due to the long waiting list, and five patients declined the MRA. Sixty-nine patients reported a previous shoulder dislocation and were not included in the analysis, as explained in the data collection section. Thus, 100 patients were analysed. Ten patients (10%) did not complete the online questionnaire.

The patient demographic characteristics are presented in Table 1. The average time between the clinical tests and the MRA was 38 days. In two cases, the two reviewers could not reach a consensus on interpretation of the images, and the second radiologist made the final diagnosis. There were no adverse events related to the MRA. Thirty-eight patients were diagnosed with a RC tear (Table 2). Among patients in the RC tear group, some were also diagnosed with traumatic anterior shoulder instability (*N* = 2), biceps pathology (*N* = 7), and superior labrum tears from anterior to posterior (SLAP; *N* = 4). In the no-RC tear group (*N* = 62), patients were diagnosed with traumatic anterior shoulder instability (*N* = 5), biceps pathology (*N* = 1), SLAP (*N* = 6), and impingement syndromes (*N* = 7). Thus, some patients had multiple diagnoses. In 45 patients, we could not find an explanation for the shoulder complaints based on the MRA.

**Table 1:** Demographic characteristics of patients with shoulder complaints

		**Baseline**	**RC group**	**No-RC group**
Number of patients		100	38	62
Mean age, years (SD)		44 (15.1)	52 (14.2)	39 (13.5)
Gender	M	65 (65%)	28 (74%)	37 (60%)
	F	35 (35%)	10(26%)	25 (40%)
Side	R	60	21	34
	L	29	8	21
	B	1	1	0
Simple Shoulder Test^a^	Yes	84%	77%	89%
	No	16%	23%	11%
Oxford Shoulder Score^b^	No night pain	16%	11%	19%
	Night pain	84%	89%	81%

*M* male, *F* female, *L* left, *R* right, *B* both. ^a^Higher score is considered a better functioning of the shoulder. ^b^Lower score is considered a better functioning of the shoulder.

**Table 2:** The different types of rotator cuff tears

**Tendon**		**Number**
Supraspinatus	Partial tear	18
	Full thickness	18
Infraspinatus	Partial tear	2^a^
	Full thickness tear	7^a^
Subscapularis	Partial tear	2^a^
	Full thickness tear	4^b^

^a^All these patients also had a supraspinatus tear. ^b^Three patients also had a supraspinatus tear, and one patient had isolated tears (traumatic).

### History information

In the cohort, 83% of the patients experienced night pain and 41% weakness of the shoulder musculature. The diagnostic value of night pain and weakness for diagnosing rotator cuff tear is presented in Table 3.

**Table 3:** Evaluations of diagnostic of history information

	**Sensitivity**	**Specificity**	**PPV**	**NPV**	**LR (+)**	**LR (−)**	**Overall accuracy**
Weakness	34.3	54.5	32.4	56.6	0.75	1.2	46.6
Night pain	88.8	19.3	41.0	73.3	1.1	0.58	46.2

Values represent percentages (%). *PPV* positive predictive value, *NPV* negative predictive value, *LR +* likelihood ratio for positive test, *LR −* likelihood ratio for negative test.

### Individual clinical shoulder tests

The diagnostic results of the nine individual clinical tests are presented in Table 4. The empty can test was the most sensitive (68.4%), the drop arm test and the lift-off test had the highest specificity (100%), and the Neer test had the best overall accuracy (75%).

**Table 4:** Evaluations of diagnostic clinical tests

	**Sensitivity**	**Specificity**	**PPV**	**NPV**	**LR (+)**	**LR (−)**	**Overall accuracy**
Empty can	68.4	56.6	49.1	74.5	1.57	0.56	75.0
Neer	63.2	82.3	68.6	78.5	3.56	0.45	61.0
Hawkins-Kennedy	52.6	77.4	58.8	72.7	2.33	0.61	68.0
Drop arm	5.3	100.0	100.0	63.3	∞	0.94	64.0
Lift-off test	13.2	100.0	100.0	65.3	∞	0.87	67.0
Painful arc	39.5	83.9	60.0	69.3	2.44	0.72	67.0
ERLS	13.2	98.4	83.3	64.9	8.2	0.88	66.0
Drop sign	5.3	100	100	63.3	∞	0.94	64.0
ISMST	15.8	98.4	85.7	65.6	9.78	0.86	67.0

Values represent percentages (%). *PPV* positive predictive value, *NPV* negative predictive value, *LR +* likelihood ratio for positive test, *LR−*: likelihood ratio (for negative test), *ERLS* external rotation lag sign, *ISMST* infraspinatus muscle strength test. ∞ = infinity.

### Prediction model

**Figure 2:**
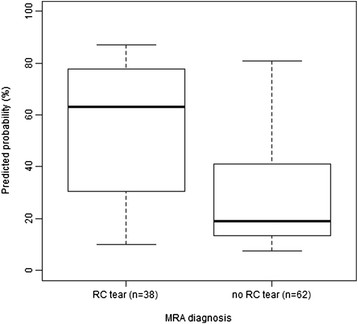
**Patient grouping and estimated probabilities of a RC tear.** Rotator cuff (RC) tears or no RC tears were diagnosed according to magnetic resonance arthrography and compared to the predicted probabilities of a RC tear, based on the prediction model. The *X*-axis represents the patients diagnosed with a RC tear or with no RC tear, according to magnetic resonance arthrography. The *Y*-axis represents the predicted probabilities of a RC tear according to the prediction model. The *box* represents graphically 50% of the patients; the *heavy line* inside the box is the median.

Of the seven preselected candidate predictors, two remained in the prediction model as independent predictors for a rotator cuff tear: age and the Neer test. The combination of clinical tests did not provide additional diagnostic value. Table 5 shows a simplified score chart that illustrates predictions from the final model after internal validation. The discriminative ability (AUC) of the model was 0.73. The ‘Hosmer-Lemeshow goodness-of-fit’ test was not significant, which indicated a good fit of the model to the data. In Figure 2, the patients were grouped according to the reference standard results into the RC tear group or the no-RC tear group; then, we plotted the estimated probabilities of a RC tear, according to the prediction model. According to our prediction model, the median probability of a RC tear was 63% in the RC tear group and 19% in the no-RC tear group.

**Table 5:** Estimations of the probability of a RC tear

**Age group**	**Negative Neer (%)**	**Positive Neer (%)**
20	14	35
30	19	44
40	25	52
50	32	61
60	40	69
70	49	76

Based on patient age (years) and the Neer test result, according to the validated prediction model. The diagnostic odds ratios of the model were as follows: older age (per 10 years), 1.42; positive Neer test, 3.32.

## Discussion

This study evaluated the diagnostic value of individual clinical shoulder tests for RC tears. In addition, we developed a prediction model that combined patient characteristics, history, and clinical test scores to improve the overall diagnostic value for RC tears.

Out of the history parameters, we found that weakness has no diagnostic value and night pain has only limited diagnostic value for rotator cuff tear. The individual clinical tests had moderate sensitivity and specificity, and overall accuracies ranged from 61% to 75%. No single test had a good discriminative value. Our hypothesis was confirmed: the prediction model, which included higher age and a positive Neer test, clearly improved the diagnostic value to detect a RC tear. However, even with our prediction model, the prediction value for rotator cuff tear is relatively low, as we were not able to develop a strong prediction model comparable to our traumatic anterior shoulder instability study [2]. Therefore, this study supports the low threshold for additional diagnostic tests for diagnosing rotator cuff tears.

A few clinical tests have a very high specificity—the drop arm, lift-off test, external rotation lag sign, and infraspinatus muscle strength test—which was also found by Bak et al. [32]. This could suggest that these are very useful clinical tests. However, because of the low incidence of a positive test result (two to seven times in this study), they seem to be less useful as a general screening tool for RC tears. However, if the test is done and it is positive, you can be almost sure that you will find a rotator cuff tear on the MRA.

Our prediction model performed well and had an adequate discriminative ability (AUC 0.73). Table 5 aims to serve as a simplified score chart that illustrates estimations of the probability that a patient will have a RC tear. After external validation, such a score could potentially be useful in deciding whether a MRA would provide added value for diagnosing rotator cuff tear.

Our prediction model has the potential for being implemented in clinical practice because it contains only two clear prediction factors. The patient's age is simply determined, and the Neer test is one of the easiest clinical tests to perform. Even without the use of lidocaine injection in the subacromial space, as originally described by Neer, the test performed very well in our prediction model. The previous study of Henkus et al. showed that it was difficult to place the injection exactly in the subacromial space, and for this reason, they considered the Neer test in combination with the injection a poor diagnostic tool [33]. Therefore, we recommend performing the Neer test without the injection.

For the development of a prediction model, it is recommended that, for each potential important predictor studied, at least five events (in this study, patients with a RC tear) are required to avoid the risk that overestimation might become problematic [34],[35]. Therefore, we assessed the prognostic value of seven predetermined potentially important predictors. The fact that our prediction model showed a strong effect of age on the probability of a RC tear was consistent with findings in the literature. RC tears have been described as a degenerative condition that increases linearly with age [32],[36].

Most previous studies evaluated these clinical tests for RC tears with arthroscopy as reference standard. That type of study design can induce verification bias, because typically, only patients with a surgical indication were tested with the reference standard [8]. A recent meta-analysis showed that MRA was the most sensitive and specific technique for diagnosing both full- and partial-thickness RC tears, compared to native MR imaging or ultrasound scans [21]. Therefore, we chose the MRA as our reference standard and performed MRAs on all patients.

Consistent with the studies of Murrell and Walton and Park et al., we found that the combination of age and a clinical test improved the diagnostic value for rotator cuff tears [6],[7]. In contrast to their results, and in agreement with the results of Bak et al. and Hermans et al., we did not find that the combination of multiple clinical tests improved the diagnostic value [5],[32]. Our study provided additional value compared to the mentioned studies for several reasons. First, we used a rigorous study design; we attempted to replicate clinical practice by combining data on patient characteristics, history, and clinical tests in our prediction model. Second, we included every patient with a shoulder complaint that visited the outpatient clinic, and we confirmed the diagnosis with MRA as the reference standard; this strategy prevented a verification bias [8]. Third, the facts that diagnoses were made by individuals blinded to patient information and decisions were made by consensus which ensured that this study was reproducible. Finally, we used state-of-the-art methodology to develop our prediction model, and we internally validated it in our dataset.

Our study also had limitations. First, although we included 100 patients with shoulder complaint and without a history of shoulder instability, only 38 had RC tears. This small sample limited our analyses by preventing the inclusion of more potentially important predictors. Moreover, we may have included a noise variable in our final prediction model. However, the internal validation procedure showed that the predictors used in our study were robust, and the shrinkage factor was fairly high. In a larger study sample, it would be interesting to do subgroup analyses to differentiate among the different tendons of the RC. Second, the examiner was not blinded to the patient characteristics and history information when the clinical tests were performed. This could lead to a bias. Because the clinical test results might have been influenced by the history information, this might explain the high diagnostic value of the individual tests in our study. We have tried to forestall by organizing a format whereby all 25 tests were performed in a rigid fixed order, thus trying to avoid this bias as much as possible. Third, we did not investigate the inter-examiner reliability of the physical examinations. However, the publication of Johansson and Ivarson showed almost perfect inter- and intra-observer agreement in four clinical tests for rotator cuff [37]. Fourth, we did not evaluate every clinical rotator cuff test published; therefore, it is possible that other clinical tests, which we did not include, might also be good predictors. Fifth, our prediction model is correlated to finding abnormalities on MRA that fit rotator cuff tear. It is still the clinician's role to determine if the findings are clinically relevant.

Before implementing our prediction model, it must first be validated with a new cohort of patients (external validation). It is also important to stress that our prediction model was developed for orthopaedic outpatient clinic patients; therefore, it may not be generalizable to primary care. The incidence of anatomical abnormalities is much higher for patients with orthopaedic complaints than for patients examined in primary care; therefore, the probability of finding a RC tear is much higher in an orthopaedic outpatient clinic [5].

## Conclusion

This prospective cohort study showed that individual clinical shoulder tests had moderate diagnostic value for the diagnosis of RC tears. Our prediction model, which combined age and the Neer test, improved the diagnostic value for diagnosing rotator cuff tears. However, the prediction value is still relatively low. Our results reconfirm that the information from the clinical examination and history has limited predicted value for finding a rotator cuff tear. Based on the current evidence, clinicians should not overestimate the diagnostic value of history and clinical examination. We recommend a low threshold for additional diagnostic tests for diagnosing rotator cuff tears.

## Competing interests

The authors declare that they have no competing interests.

## Authors’ contributions

DAvK designed the study and wrote the protocol. He managed the database, performed the analysis, and wrote and revised the manuscript. TvdB helped with the database management, performed parts of the analysis, and reviewed the manuscript. HJvdW reviewed all the MRA together with WJW and critically reviewed the manuscript. RMC helped design the study and critically reviewed the manuscript. VABS helped design the study and critically reviewed the manuscript. CBT helped design the study, advised on the statistical analysis, and critically reviewed the manuscript. WJW helped design the study, included all the patients at the outpatient clinic, and reviewed all the MRA together with HJvdW. He also critically reviewed the manuscript. All authors read and approved the final manuscript
